# Infrared Spectroscopy Can Differentiate Between Cartilage Injury Models: Implication for Assessment of Cartilage Integrity

**DOI:** 10.1007/s10439-024-03540-x

**Published:** 2024-06-20

**Authors:** Fatemeh Shahini, Soroush Oskouei, Ervin Nippolainen, Ali Mohammadi, Jaakko K. Sarin, Nikae C. R. te Moller, Harold Brommer, Rubina Shaikh, Rami K. Korhonen, P. René van Weeren, Juha Töyräs, Isaac O. Afara

**Affiliations:** 1 Department of Technical Physics, University of Eastern Finland, Kuopio, Finland; 2 Diagnostic Imaging Center, Kuopio University Hospital, Kuopio, Finland; 3 Department of Medical Physics, Tampere University Hospital, Wellbeing Services County of Pirkanmaa, Tampere, Finland; 4 Department of Clinical Sciences, Faculty of Veterinary Medicine, Utrecht University, Utrecht, The Netherlands; 5 Centre for Radiation and Environmental Science, FOCAS Research Institute, Technological University Dublin, Dublin, Ireland; 6 Regenerative Medicine Utrecht, Utrecht, The Netherlands; 7 School of Electrical Engineering and Computer Science, The University of Queensland, Brisbane, Australia; 8 Science Service Center, Kuopio University Hospital, Kuopio, Finland

**Keywords:** Mid-infrared spectroscopy, Articular cartilage, Chondral groove model, Equine, Osteoarthritis, Machine learning

## Abstract

In order to improve the ability of clinical diagnosis to differentiate articular cartilage (AC) injury of different origins, this study explores the sensitivity of mid-infrared (MIR) spectroscopy for detecting structural, compositional, and functional changes in AC resulting from two injury types. Three grooves (two in parallel in the palmar-dorsal direction and one in the mediolateral direction) were made via arthrotomy in the AC of the radial facet of the third carpal bone (middle carpal joint) and of the intermediate carpal bone (the radiocarpal joint) of nine healthy adult female Shetland ponies (age = 6.8 ± 2.6 years; range 4–13 years) using blunt and sharp tools. The defects were randomly assigned to each of the two joints. Ponies underwent a 3-week box rest followed by 8 weeks of treadmill training and 26 weeks of free pasture exercise before being euthanized for osteochondral sample collection. The osteochondral samples underwent biomechanical indentation testing, followed by MIR spectroscopic assessment. Digital densitometry was conducted afterward to estimate the tissue's proteoglycan (PG) content. Subsequently, machine learning models were developed to classify the samples to estimate their biomechanical properties and PG content based on the MIR spectra according to injury type. Results show that MIR is able to discriminate healthy from injured AC (91%) and between injury types (88%). The method can also estimate AC properties with relatively low error (thickness = 12.7% mm, equilibrium modulus = 10.7% MPa, instantaneous modulus = 11.8% MPa). These findings demonstrate the potential of MIR spectroscopy as a tool for assessment of AC integrity changes that result from injury.

**Supplementary Information:**

The online version contains supplementary material available at 10.1007/s10439-024-03540-x.

## Introduction

Articular cartilage (AC) is a specialized connective tissue covering the surface of bones in diarthrodial joints [1]. It plays a crucial role in joint function, providing a smooth and lubricated surface for movement [2]. AC is composed of a complex extracellular matrix (ECM) that includes water, collagen, PGs, and other macromolecules [3]. These include non-collagenous proteins, such as fibromodulin, albumin, fibronectin, decorin, and biglycan, which play crucial roles in maintaining the tissue's biological and mechanical properties, as well as in chondrocyte-matrix interactions [4]. However, due to its avascular and aneural nature, AC has limited intrinsic regenerative capacity, and injury to this tissue can result in further degeneration, often resulting in joint pain, stiffness, and functional impairment [5]. AC degeneration leads to alteration of its composition, structure and integrity, which are significantly influenced by molecular factors, such as matrix metalloproteinases (MMPs). In particular, MMP-1 and MMP-13, play crucial roles in the breakdown of collagen and the degradation of ECM [6, 7], driving the degenerative processes that lead to osteoarthritis (OA); OA is a progressive condition that mostly impacts the AC but also affects other joint components such as the synovial membrane and subchondral bone [8].

Clinical diagnosis of AC injury involves several methods, including physical examination and imaging (computed tomography and magnetic resonance imaging), with arthroscopy used as a means for assessing the severity and extent of injury during surgical intervention. This surgical procedure involves the insertion of an endoscope into the joint space to directly visualize and assess the AC surface [9]. However, it has several limitations, including subjectivity, poor reproducibility, and lack of sensitivity to early-stage AC degeneration [10].

Animal models of OA have been extensively used to understand the pathophysiology of AC degeneration [10–12] due to the limited availability of human samples. Studies have demonstrated that, of the species examined, equines are the most similar to humans with respect to AC thickness in the stifle (knee) joint [13, 14]. Their cellular structures, biochemical compositions, and biomechanical characteristics closely resemble those found in humans [15]. Recent animal studies have demonstrated the potential of the articular groove model for evaluating degenerative joint changes caused by chondral injuries [16–18]. This model has the potential to enhance our understanding of AC injuries, particularly at the early stages where visual assessment is subjective, and provide insight into the development and management of OA. However, available clinical techniques based on arthroscopy for diagnostic assessment of the joint injuries during treatment are unable to effectively detect these early stages injuries due to their subjective nature.

Optical methodologies, such as optical coherence tomography [19, 20], near-infrared (NIR) [21, 22], MIR [23, 24], and Raman spectroscopy [25, 26], have emerged as promising minimally invasive tools to assess the biochemical composition and quality of AC. Of these methods, MIR spectroscopy has been increasingly used due to its sensitivity to changes in collagen and PG content [27], two important components of the cartilage ECM that contribute to its mechanical properties [1]. The strengths of MIR spectroscopy lie in its ability to provide better molecular sensitivity than NIR spectroscopy, as it is based on fundamental molecular vibrations [28]. Moreover, MIR spectroscopy has shown promising results for predicting AC mechanical properties [29]. Therefore, this technique can potentially serve as a valuable tool for minimally invasive assessment of different types of AC defects based on distinct changes in the spectral signature of collagen and PGs. In MIR spectroscopy, the measurement focuses on how MIR light (4000–400 cm^−1^) interacts with the sample. The spectrum obtained from this process reveals insights on the molecular composition and structure of the sample [30]. Along with MIR, OMIC-based technologies such as Nuclear Magnetic Resonance (NMR) and Mass Spectrometry (MS) are gaining attention [31]. NMR provides detailed structural insights, whereas MS excels at sensitive biomarker detection. Despite this, each technique has its specific applications and limitations, needing a comprehensive approach. MIR spectroscopy presents various advantages over NMR and MS, including non-destructive analysis, minimal sample preparation, quicker data acquisition, cost-effectiveness, high sensitivity to functional groups, and ease of use, making it a viable option for rapid and efficient molecular analysis [32]. By demonstrating the capability of MIR spectroscopy to identify subtle molecular changes in AC, this study aims to contribute to the advancement of diagnostic methods in clinical settings, particularly for the early detection and effective treatment of AC injuries and diseases.

The MIR spectral region consists of fundamental molecular vibrations, and interpreting the resulting spectrum of AC can be challenging due to the complex and overlapping spectral features of its constituents. Hence, direct assessment of specific tissue properties, such as collagen content, based on the MIR spectrum alone is challenging. However, machine learning (ML) techniques can aid in establishing relationship between the MIR spectrum and the tissue’s properties, enabling effective and accurate tissue characterization.

In this study, we hypothesize that MIR spectroscopy is sensitive to changes in AC structure and composition consistent with early stages of OA induced via an animal model of OA. By incorporating ML techniques, we hypothesize that MIR spectroscopy can differentiate between healthy tissue and different types of AC injuries and accurately estimate their structural, compositional, and functional properties. To test this hypothesis, we collected MIR spectra from healthy and injured equine AC (the injury was induced using OA models [33]). We then developed ML models to classify the samples based on their MIR spectra, as well as to estimate the tissue's properties (e.g., thickness, composition and mechanical properties) from the MIR spectra of the samples.

## Materials and Methods

**Fig. 1:**
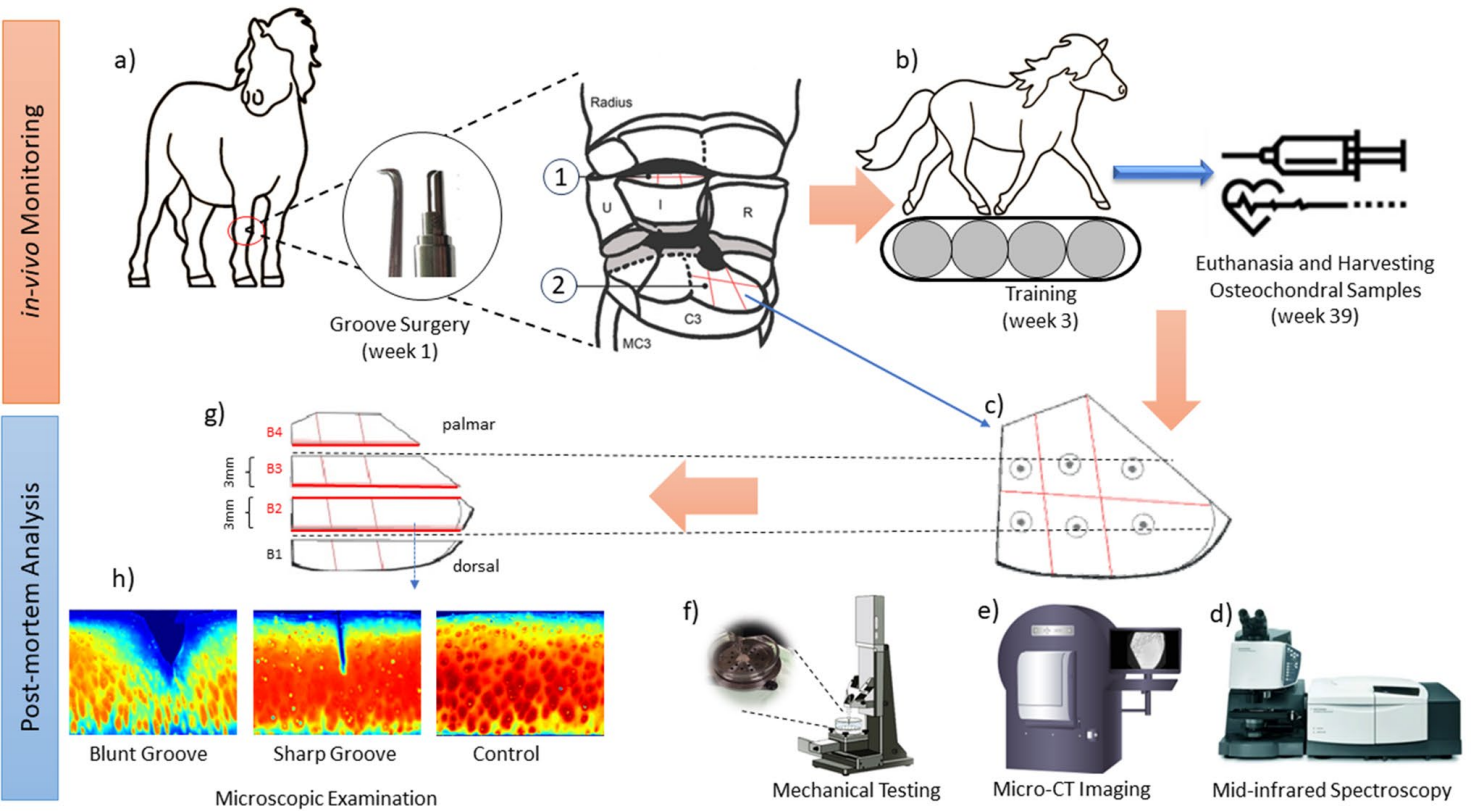
Complete flow diagram of the measurement protocol along with sample extraction locations. **a** Blades used for surgery [blunt (left) and sharp (right) grooves], the carpal groove model is shown for the right carpus, with the left carpus representing the sham‐operated control (red circle). Grooves were created at the dorsoproximal surface of the intermediate carpal bone (1) and at the radial facet of the proximal surface of the third carpal bone (2). **b** The exercise program and harvesting samples, **c** locations used for spectroscopic measurement and mechanical indentation tests, **d** mid-infrared spectroscopy, **e** micro-CT imaging, **f** mechanical testing, **g** preparing the tissue sections for histological analyses (the bold red lines indicate locations for harvesting the sections), and **h** microscopical analysis (digital densitometry) as well as representative images of PG content in control, sharp, and blunt grooved cartilage

Nine healthy adult female Shetland ponies (mean ± SD) age 6.8 ± 2.6 years (range 4–13 years); were utilized in this study, originally designed to quantify the long‐term progression of blunt and sharp AC defects and their impact on joint homeostasis and function in the equine carpus [34]. The number of ponies was chosen after a power analysis (power 0.9 and *p* < 0.05) based on a pilot study [33] and other previous groove model studies [16, 33, 35]. The duration and design of the exercise protocol were adapted for Shetland ponies from an existing training protocol for horses in a different AC defect model with a similar study set-up. The training intensity was based on a balance between being challenging enough to ensure increased joint loading and being mild enough to avoid the animals getting lame. The protocol was tested extensively in a separate pilot study prior to the groove model experiment. Each defect consisted of three grooves (two in parallel in palmar-dorsal direction and one in mediolateral direction), which were made via arthrotomy in the AC of the radial facet of the third carpal bone (middle carpal joint) and of the intermediate carpal bone (the radiocarpal joint), as described previously [36, 37] (Fig. 1a). Blunt and sharp defects were randomly assigned to each of the two joints. The sharp method causes minimal tissue damage (mild AC degenerative phenotypes), while the blunt method results in more significant damage (moderate AC degenerative phenotypes), including tissue loss and potential damage to the calcified AC layer [34]. The distal surface of the radius and the distal surface of the radial carpal bone were denominated as kissing sites, where the groove was in contact with the opposing AC. In clinical practice, the term "kissing site" refers to the area of a joint that is in contact with a lesion [38]. When a lesion is present, it can rub against the normal, unaffected joint surface, causing damage. This was observed in an experiment where blunt lesions were created on the joint surface. In most samples from blunt‐grooved joints, an imprint was seen indeed at the kissing site. The kissing-blunt is the contact site of bluntly grooved samples. The kissing-sharp is the contact site of sharply grooved samples. The damaged group includes all the grooved (blunt, *n* = 9; sharp, *n* = 9) and kissing grooved samples (*n* = 18). The contralateral joints were sham-operated without any manipulation of the AC and used as healthy control joints.

After 3 weeks of box rest, the ponies were trained for 8 weeks on a treadmill (Fig. 1b). Pony training on a treadmill was conducted as part of the study to assess the gait pattern changes and biomechanical responses following the surgical procedures involving blunt and sharp grooves in the articular cartilage. The treadmill training allowed researchers to evaluate the ponies' locomotion, gait alterations, and biomechanical responses under controlled conditions. After completing the 11-week exercise protocol, the ponies were allowed to roam freely in the open group shed (approximately 125 m^2^). After 26 weeks, they were allowed free pasture exercise. At 39 weeks, the ponies were sacrificed, and both grooved and control joints were harvested. The joints were stored at − 20 °C. Later, the samples were thawed in a water bath at 4 °C and osteochondral samples were harvested from grooved and kissing sites in both grooved and contralateral joints and stored at − 20 °C for the following experiments (Fig. 1d–f).

### MIR Spectroscopy Measurements

All osteochondral samples were scanned using an Agilent Cary 670/620 FTIR imaging spectrometer (Agilent Technologies, Inc) equipped with a 128 × 128 focal plane array (FPA) detector with a spectral range of 3800–800 cm^−1^ (Fig. 1d). Each point measurement was acquired from a 100 × 100 µm filter area in reflection mode using a × 15 Cassegrain objective, with pixel size of 5.5 µm, spectral resolution of 4 cm^−1^, 64 coadded scans, and penetration depth of 5–10 µm. Non-contact MIR spectroscopy was performed on cartilage surfaces at six points per sample, with three repeats per point, totaling approximately 35 min of exposure per sample. To mitigate water loss, we conducted measurements in a humidity-controlled environment by keeping the cartilage surface hydrated with a saline-soaked gauze, and only exposing the point being measured with MIR. These steps ensured the preservation of sample integrity and the reliability of our spectral data analysis. The average spectrum was used for analysis. It is worth noting that the resulting measurements were spectral data and not images (2D or cube). The spectral data were directly acquired from the samples using MIR spectroscopy via a microscope objective. Thus, the spectrum of each sample is represented by 1-dimensional wavelength-dependent absorption values. The collected spectral data were then compiled in CSV format. Before machine learning analysis, the data underwent spectral preprocessing steps, such as normalization, noise reduction to enhance the quality and consistency of the data, and subtraction of a reference background spectrum from the signals to minimize the impact of atmospheric humidity on entire spectral data.

### Thickness Measurements and Mechanical Indentation Test

Osteochondral samples were thawed overnight at 4 °C and subsequently brought to room temperature before micro-CT imaging. The joints were at room temperature between the scans. Osteochondral samples were imaged with a high resolution micro‐CT imaging (Quantum FX®; Perkin Elmer) in order to estimate the AC thickness, which is also required for mechanical indentation testing. To ensure the preservation of their native hydrated state during the Micro-CT process, the samples were stored in sealed containers during measurements in order to maintain a humid environment, and this was achieved by placing wet tissues inside the containers. This measure was taken to closely mimic physiological conditions and prevent dehydration that could potentially affect the imaging quality and accuracy. A detailed protocol for the estimation of AC thickness using this approach is presented elsewhere [16]. Subsequently, samples were stored at − 20 °C until the mechanical indentation test.

Six measurement locations were selected on the surface of thawed samples (Fig. 1c). In grooved samples, three points were chosen on the dorsal and palmar side of the groove, running mediolaterally. The locations were the same for kissing sites and contralateral control samples based on an estimated virtual groove [16, 34, 37]. Samples were glued to the bottom of a measuring chamber for each measurement, and the chamber was filled with phosphate-buffered saline (PBS). A one-step stress-relaxation protocol (the strain of 15% of AC thickness with 100%/s strain rate followed by 300s relaxation) was applied perpendicular to each of the measurement locations using a Biomomentum Mach-1 v500css (Biomomentum Inc., Laval, Quebec, Canada) device with a 70 N multiaxial load-cell and a non-porous spherical tip indenter (*d* = 0.5 mm, MA034, Biomomentum).[16] The estimated equilibrium and instantaneous Young's moduli from equilibrium and peak stress/strain ratios were corrected using the Hayes equation[39], with the use of Poisson ratios of *ν* = 0.2 [40] and *ν* = 0.5 for the equilibrium and instantaneous modulus, respectively [40–42]. All specimens were stored in PBS in between the mechanical testing and MIR measurement, to avoid dehydration.

### Microscopic Examination

After MIR spectroscopy and biomechanical measurements, the osteochondral samples were cut at 6 mm along the central groove towards the dorsal and palmar sides. Subsequently, they were fixated in 10% formalin (Riedel‐de Haen 33220) and decalcified in 0.5 M ethylenediaminetetraacetic acid (prod. 20296.360, VWR; Radnor) at pH 7.0 for 10 weeks. After decalcification, the samples were cut into 4 parts (Fig. 1g), dehydrated in graded ethanol solutions, and embedded in paraffin. The cutting lines B1–B2 and B3–B4 were along the points where biomechanical and MIR measurements were performed. Three adjacent 5 µm thick sections from the locations corresponding to the biomechanical indentation test points were cut with a microtome and stained with Safranin-O/Fast-Green.

Digital densitometry (DD) imaging was conducted in exact accordance with the methods outlined in Mohammadi et al. study [16]. DD imaging was performed using a light microscope (Nikon Microphot FXA, Tokyo, Japan) equipped with a CCD-cooled camera [Hamamatsu Photonics K.K, Hamamatsu City, Japan, pixel size = 1.4 μm, the effective number of pixels = 1344 (H) × 1024 (V)] and $\times$ 4 magnification (Fig. 1h). Images were calibrated against neutral density filters (optical density values 0.0, 0.3, 0.6, 1.0, 1.3, 1.6, 2.0, 2.6, and 3.0) (Schott, Mainz, Germany). Three sections per injury type were examined, and utilized their average values in the spectral analysis. This approach ensures that our results account for the variability within each section and reflect a more accurate estimation of optical density measurements across the tissue. The optical density of the images (which reflects the fixed charged density of PGs [43]) was used to estimate the PG distribution in the tissue [16]. Subsequently, the spectral data of each sample plug was compared with its corresponding PG content, creating a predictor (spectra)—target (PG content) pair dataset for ML analysis.

### Data Analysis

Spectral preprocessing was conducted using *nippy* (http://github.com/uef-bbc/nippy), an open-source spectral preprocessing toolbox [44]. The preprocessing on the spectra includes the following stages: (1) clipping the data to limit it to the region of interest (800–1800 cm^−1^) due to its significant biomolecular signatures relevant to cartilage, (2) Filling missing wavenumber values using linear interpolation, (3) noise reduction using a moving average window (*L* = 7) and (4) baseline removal with a moving median filter (window = 101), (5) Outlier samples with aberrant shapes or with absorption values less than 3% or greater than 97% of the average in every wavenumber were removed from the analysis. The mean spectra of each damage and control group were calculated.

In order to investigate the relationship between the samples spectra and their properties, we employed several ML algorithms, including support vector machine (SVM), decision tree (DT), boosted tree (BT), and random forest (RF). These algorithms were used to develop models for discriminating control from damaged AC (classifiers) and estimating AC properties (regressors) based on spectral data. Classifiers were developed for discriminating between the following sample groups:

1. Control vs. grooved (blunt grooved and sharp grooved)
2. Control vs. kissing grooved
3. Blunt vs. sharp
4. Kissing blunt vs. kissing sharp
5. Grooved vs. kissing grooved
6. Control vs. damaged (blunt grooved, sharp grooved, kissing blunt, kissing sharp)

To optimize the performance of each algorithm, we carefully investigated, selected, and tuned their hyperparameters. Among these algorithms, RF and SVM resulted in the best models after hyperparameter optimizations (Table 1). Hence, only these algorithms were used for building the final models.

**Table 1:** The different machine learning algorithms employed and their corresponding hyperparameters, support vector machine (SVM), decision tree (DT), boosted tree (BT), and random forest (RF)

Method	Searched parameters	Searched values	Optimum (regressions)	Optimum (classifications)
SVM	Kernel	Linear, Poly, Rbf, Sigmoid	Poly	Rbf
	C	0.1, 1, 10	1	0.1
	Degree	1, 2, 3, 4	3	–
	Gamma	Auto, Scale	Scale	Auto
DT	Maximum_Depth	None, 2, 3, 10, 20	–	–
	Minimum _Samples_Split	2,4,6		
	Minimum _Samples_Leaf	1,3,5		
BT	Learning_Rate	0.01, 0.1, 0.3	–	–
	N_Estimators	10, 100, 200		
	Subsample	0.1, 0.2, 0.4		
RF	Maximum depth	None, 3, 10, 20, 50	None,20,50	None
	Maximum features	None, 8, 20, 50,100, 200	200	200
	Minimum samples leaf	4, 5, 7	4	2
	Minimum samples split	5, 7, 10	5	2
	Number of estimators	100, 500	100	100
	Random state	0, 10, 20, 50, 100, 200	100	300

Monte Carlo cross-validation (CV) was used to randomly select 2 ponies for training and testing the models. The classification performance (assessed via accuracy and precision) was evaluated for each of the Monte Carlo CV test sets (250 runs). The reported values are the average values of the 250 runs. For regression, Monte Carlo CV with random training (80%) and test (20%) sets were used to assess the performance of each model. CV was performed for 200 iterations, and the average root-mean-square error (RMSE) of calibration (RMSEC) and prediction (RMSEP) were determined.

## Results

**Fig. 2:**
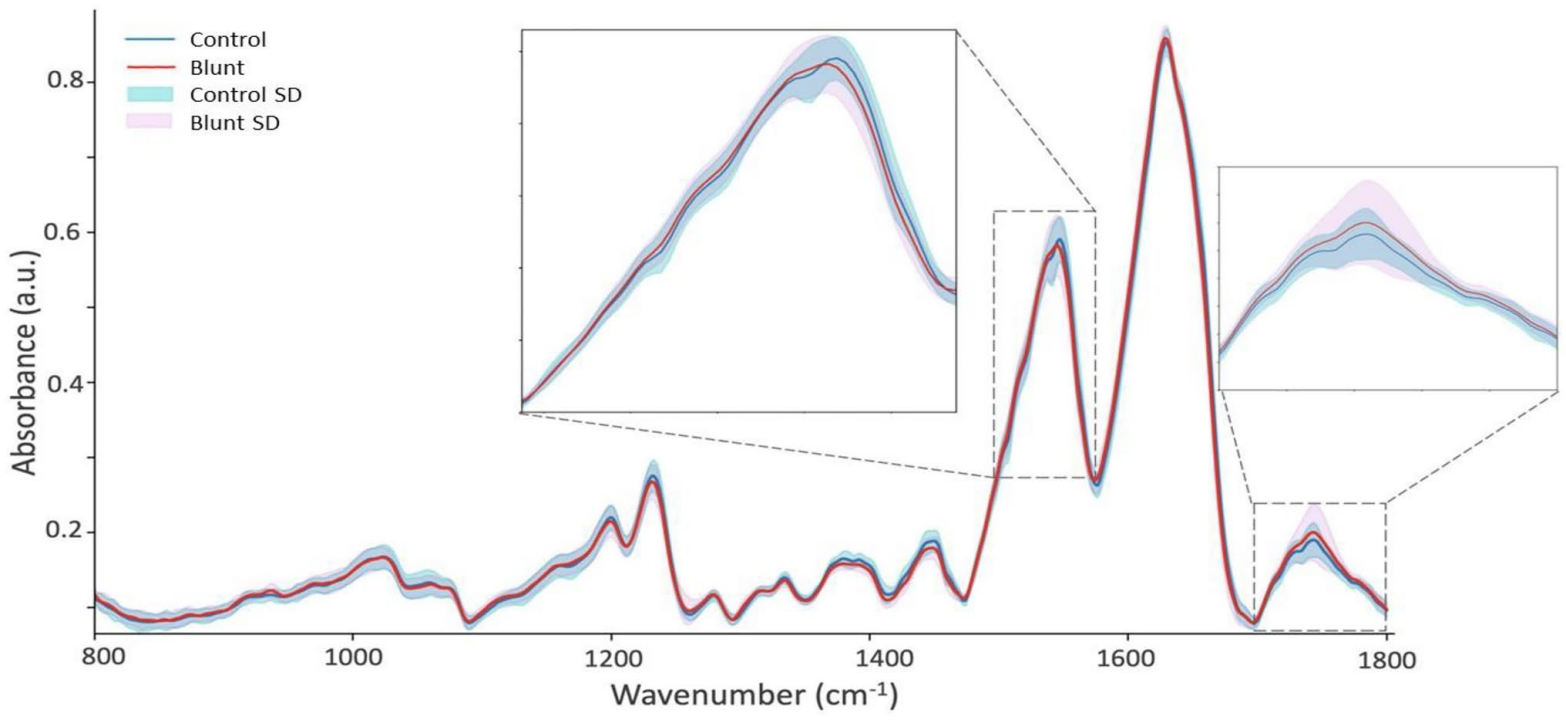
Mean spectra of blunt groups and control groups. The Blunt group includes 54 samples, while the control group includes 105 samples. The solid lines represent the mean of the values, whereas the shaded areas represent the standard deviations. Standard deviation was calculated for each group using the formula SD = square root of [(sum of (each value − mean value)^2^)/(number of values − 1)]

**Fig. 3:**
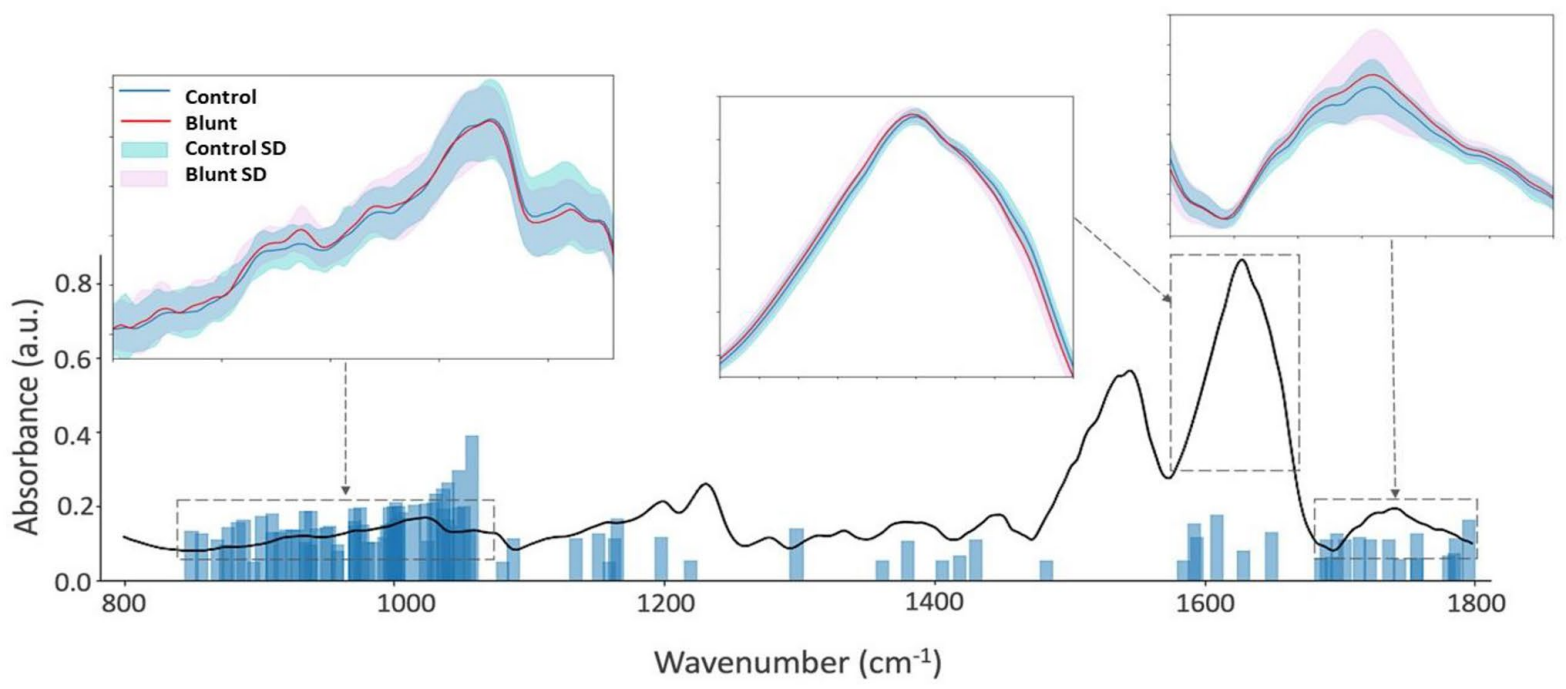
Feature importance for the selected RF model for the classification of samples of blunt groups and control groups. The solid lines represent the mean of the values, whereas the shaded areas represent the standard deviations (SD)

**Fig. 4:**
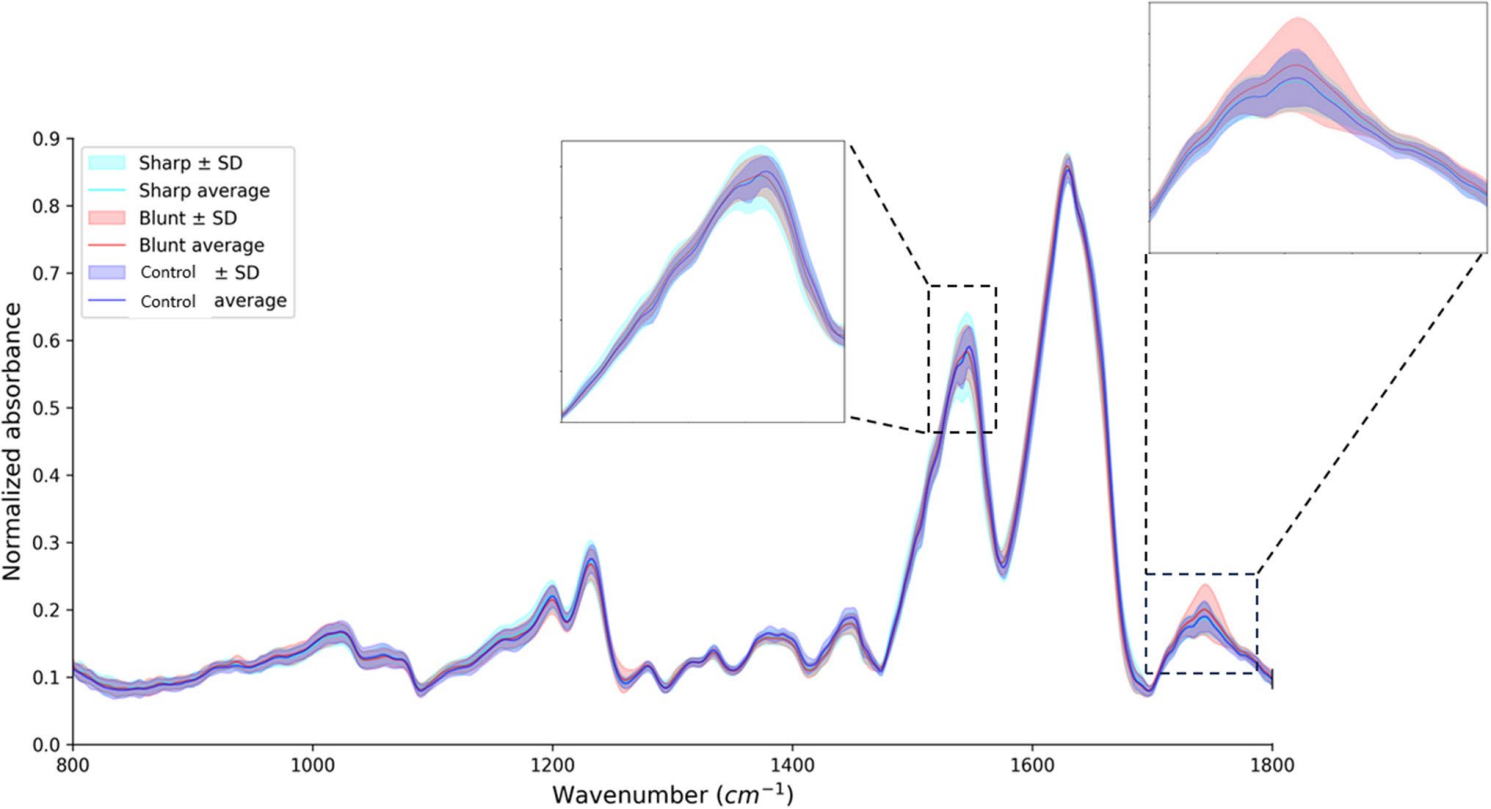
Mean spectra of the Grooved (Blunt and Sharp) group and control group. The Blunt group includes 54 samples, the sharp group includes 45 samples, while the control group includes 105 samples. The solid lines represent the average of the values, whereas the shaded areas represent the standard deviations. Standard deviation was calculated for each group using the formula SD = square root of [(sum of (each value − mean value)2)/(number of values −  1)]

**Fig. 5:**
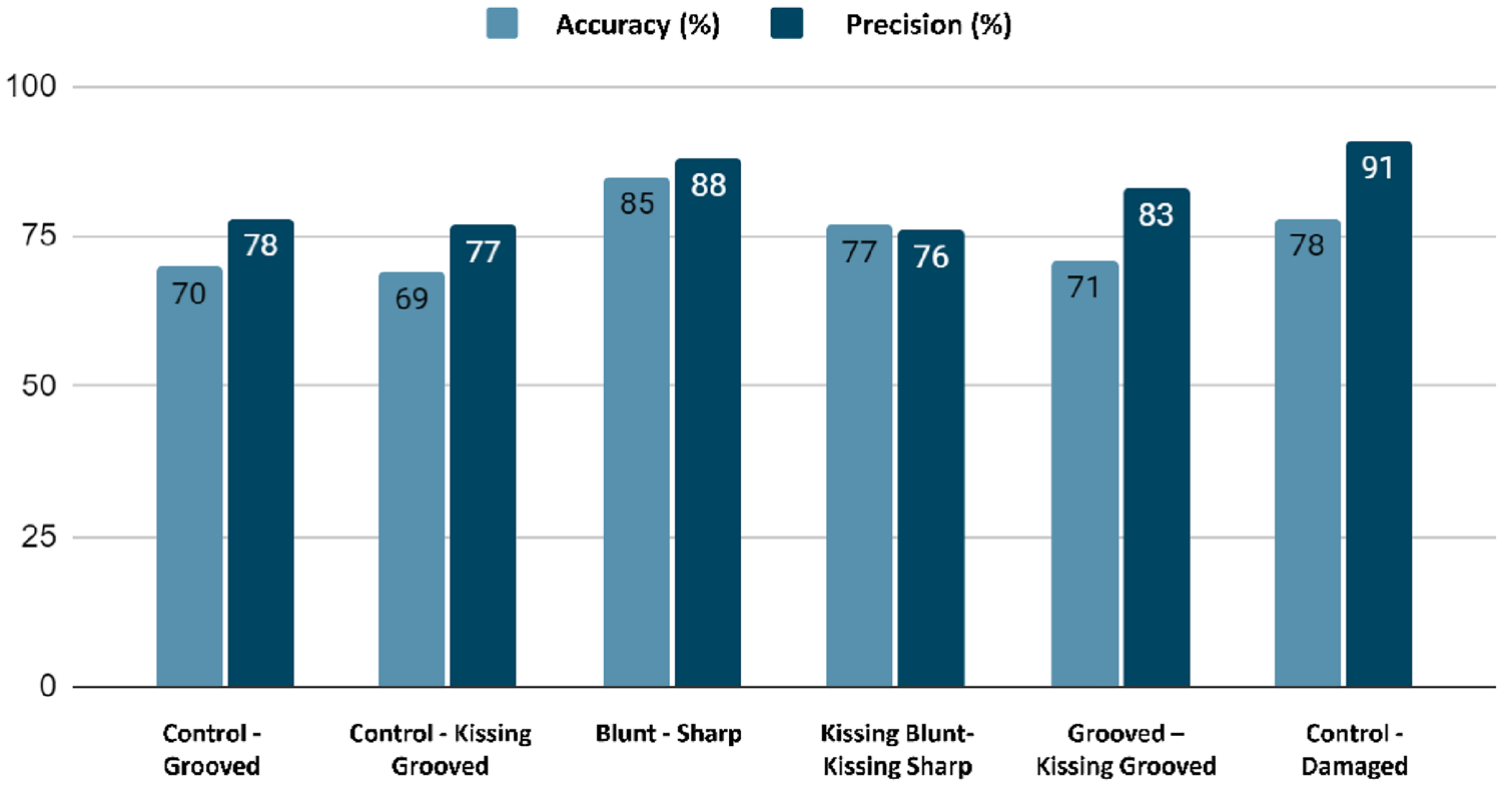
Classification performance depicted through accuracy and precision scores derived from machine learning models using a Monte Carlo cross-validation process on cartilage spectral data. Accuracy measures the model's overall correct classifications, combining true positives and true negatives. Precision assesses the model's correctness in identifying damaged tissue, focusing on minimizing false positives. High values in these metrics indicate a reliable model in correctly diagnosing tissue state, crucial for clinical applications

In our study, MIR spectroscopy was utilized to investigate the surface and near-surface biochemical changes in AC, with a focus on proteoglycan content. Given the penetration depth of approximately 5–10 µm for MIR radiation, our analysis predominantly reflects the molecular composition of the cartilage's superficial layers. Despite this limitation, the surface-level changes detected by MIR spectroscopy offer significant insights into the overall health of the cartilage. The results reveal differences between the MIR spectra of control and grooved (blunt and sharp grooves) cartilages. The mean spectra of the blunt group exhibited notable differences compared to the control group over the other damaged groups (Fig. 2), particularly in the Amide $\text{\rm I}$ (1580–1720 cm^−1^, Amide $\text{\rm I}\text{\rm I}$ (1490–1580 cm^−1^), and Ester carbonyl (1700–1750 cm^−1^) regions. However, the kissing sites were found to exhibit only marginal differences in comparison to the mean spectra of the control groups, particularly in the absorbance peaks of Amide $\text{\rm I}$, Amide $\text{\rm I}\text{\rm I}$, and Ester carbonyl regions, when comparing the mean to variation of spectra in the grooved groups (Fig. 3**). The main feature observed in the MIR spectrum of the damaged AC groups (blunt and sharp groups) is an overall increase in absorbance across the spectral region (Fig. 4). Both groove groups also show different spectra profiles compared to the control group. The absorbance peaks corresponding to collagen in the range of 1080–1458 cm^−1^, along with the presence of C=O stretching at 1740 cm^−1^ (Figs. 3, 4), emerged as crucial features in the feature importance analysis (Fig. 5), validating previous studies [45] that highlighted the significant role of this collagen range in distinguishing between healthy (control) and damaged AC.

The performance of the classification models is visualized in a grouped bar chart in (Fig. 5). The control group was compared to the grooved groups (blunt grooves and sharp grooves), the kissing grooved group (kissing-sharp grooved and kissing-blunt grooved), the bluntly grooved group, the sharply grooved group, and the damaged group, which contains the blunt, sharp, kissing-blunt, and kissing-sharp groups. The performance of the different classifiers for discriminating control from damaged AC shows moderate to high accuracy and precision (Fig. 5). The highest accuracy rate(the model's overall success in correctly classifying samples into blunt and sharp injury types) was observed in the model discriminating between the blunt and sharp grooved group (85%), while precision rate(model's ability to correctly predict the control versus damaged groups) was the highest (91%) in the model discriminating between the control and the damaged group.

**Fig. 6:**
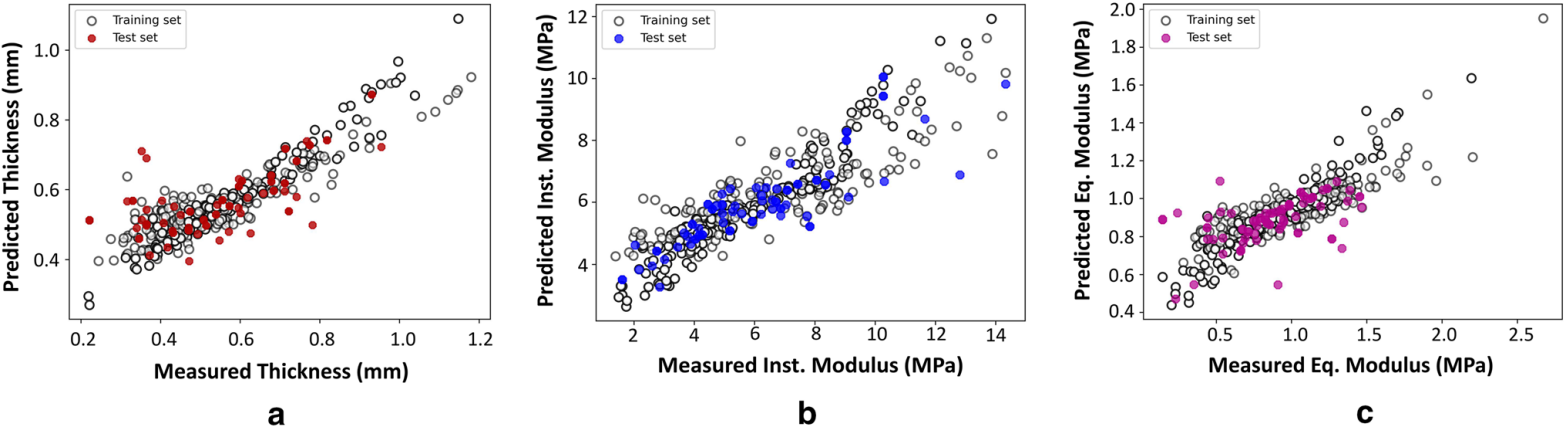
Correlation between the measured and MIR-predicted values obtained with the best models developed for **a** thickness, **b** instantaneous modulus, and **c** equilibrium modulus using MIR spectral data. The horizontal axis represents the predicted values, while the vertical axis represents the true values. The blue dots and red crosses correspond to the test and training sets, respectively. These plots provide a visual representation of the accuracy of the predictions, with a perfect prediction resulting in a diagonal line

This study obtained the mechanical indentation test and full thickness PG content data utilized for developing the machine learning models as part of an earlier study [16]. The specific data are not reported here in detail. The performance of the regression models for estimating thickness, instantaneous modulus, and equilibrium modulus from the MIR spectra (Fig. 6) demonstrate strong relationships between the spectra and AC functional properties as shown by the low RMSEP values between the predicted and true properties (Table 2).

**Table 2:** Partial least squares regression (PLSR) model performance for estimating cartilage biomechanical properties for control and damaged groups

Target	RMSEC	RMSEP	RMSEP (%)	MAE	MAE (%)
Thickness (mm)	0.073	0.122	12.7	0.054	9.2
Equilibrium (MPa)	0.270	0.270	10.7	0.194	7.7
Instantaneous (MPa)	1.582	1.528	11.8	1.181	9.1

Mean absolute error (MAE), root mean square error of calibration (RMSEC), root mean square error of prediction (RMSEP)

**Fig. 7:**
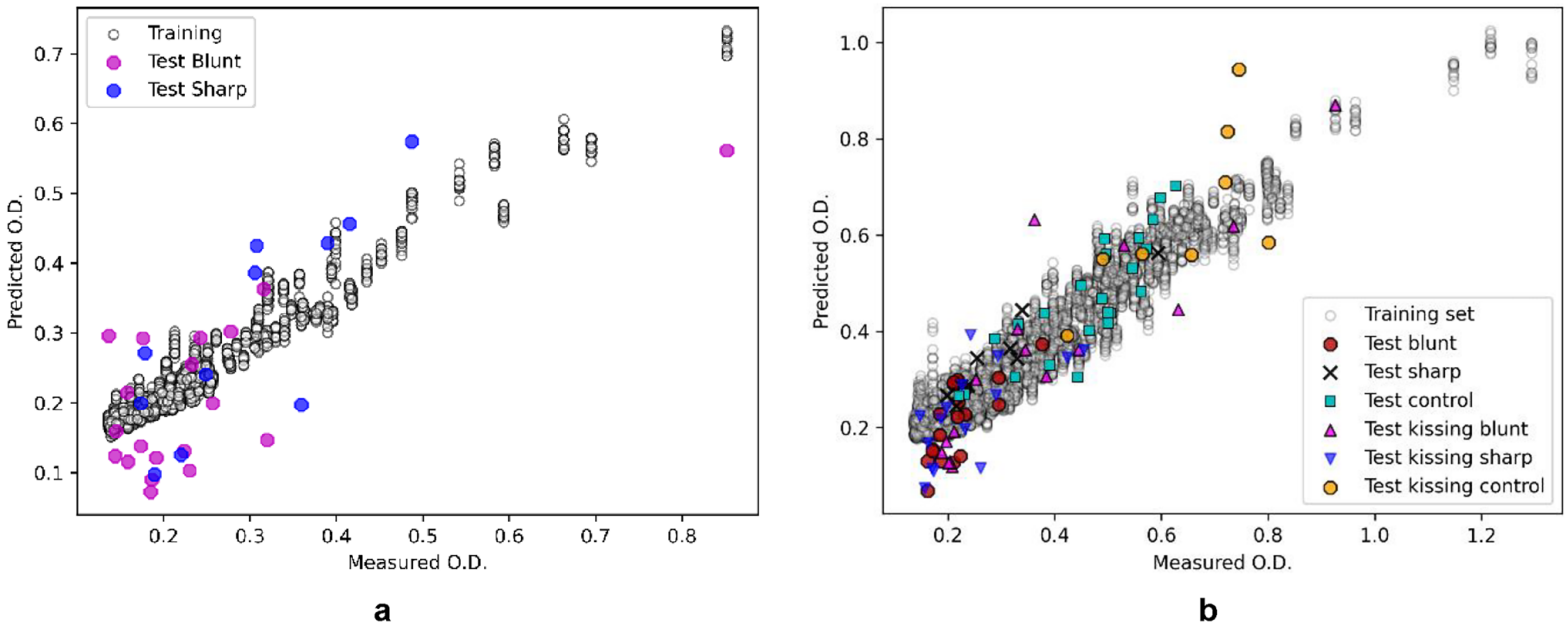
The relationships between measured and predicted cartilage full-thickness PG content. **a** The grooved group. **b** All the groups

To quantitatively assess the relationship between spectral features and proteoglycan content, machine learning models were developed. These models analyzed MIR spectral data, enabling the prediction of cartilage full-thickness PG content. Plots comparing measured versus predicted PG content effectively highlight the correlation between surface-level spectral signatures and deeper tissue properties. The regression models for estimating the samples' PG content also demonstrate a strong relationship between the samples' MIR spectra and their PG content determined via DD (Fig. 7), with relatively low error (Tables 3, 4). The models exhibit low error in both scenarios of training with only the damaged group (specific model—Fig. 7a) and with the entire sample set that includes the control and damaged groups (generalized model—Fig. 7b).

**Table 3:** PLSR model performance for estimating cartilage full-thickness PG content for grooved groups. Mean absolute error (MAE), root mean square error of prediction (RMSEP)

Full-thickness PG content	RMSEP	RMSEP (%)	MAE	MAE(%)
Bluntly Grooved Group	0.108	15.1	0.085	11.9
Sharply Grooved Group	0.087	27.9	0.076	24.4

**Table 4:** PLSR model performance for estimating cartilage full-thickness PG content for all groups. Mean absolute error (MAE), root mean square error of prediction (RMSEP)

Full-thickness PG content	RMSEP	RMSEP (%)	MAE	MAE(%)
Bluntly Grooved Group	0.055	25.6	0.045	20.9
Sharply Grooved Group	0.062	15.7	0.055	14.0
Control Group	0.067	15.2	0.060	13.6
Kissing Blunt	0.103	8.4	0.081	7.5
Kissing Sharp	0.077	20.1	0.067	18.0
Kissing Control	0.116	16.4	0.089	14.7

## Discussion and Conclusions

In the present study, we demonstrated the capacity of MIR spectroscopy for assessing compositional and functional changes in AC resulting from different types of injury. The injury models used were aimed at mimicking mild to moderate AC damage, and the degenerative changes from these models are similar to those observed in OA [17, 18]. The results here could assist in detecting compromised AC prior to symptoms of matrix degeneration. The results demonstrate that this optical approach can classify samples according to the severity of the injury. This suggests that the findings in this study could be translated to the characterization of OA-induced AC damage and that MIR spectroscopy could provide insights into biomolecular tissue-level changes during the development and progression of (early) OA.

Absorption in the MIR spectral range arises mainly from fundamental vibration of chemical bonds such as O–H, N–H, CH, C=O, C–N, C–O, and PO bonds, which are abundant in biological tissues. This makes MIR spectroscopy ideal for detecting and characterizing biochemical changes in AC during injury, providing valuable information that is comparable to results obtained from methods such as DD. More so, the sensitivity of MIR spectroscopy to AC biomechanical properties is likely due to the structure-function coupling of its matrix, where changes in the biochemical composition and structure of the AC extracellular matrix are reflected in its biomechanical properties [24, 27, 46]. Thus, the features embedded in the MIR spectrum, which contains information on important physicochemical [46, 47] and morphological properties of the tissue [48, 49], are the likely reason for the capacity of MIR spectroscopy for discriminating normal from damaged AC, as well as estimating the tissue's composition and functional properties.

The MIR spectral analysis of articular cartilage (AC) post-injury indicated discernible molecular alterations, especially within the Amide I (1580–1720 cm^−1^) and Amide II (1490–1580 cm^−1^) regions. These regions are reflective of changes in the protein content, possibly alterations in the collagen's secondary structure. Additionally, variations observed in the ester carbonyl region (1700–1750 cm^−1^) suggest disruptions in the content or organization of lipid components within the extracellular matrix (ECM), or differences in lipid content in the joint space. It is important to note that the increase in absorbance at 1740 cm^−1^, often attributed to C=O stretching, may also signify changes beyond collagen and lipid alterations and point towards a matrix-wide degradation. These observations underscore the complexity of spectral changes and the possibility that they may also reflect contributions from lipid content in the joint space. The observed variations in the broader spectral region of 1080–1458 cm^−1^, which encompasses absorbances from collagen, further underscore the complex impact of injury on AC molecular composition. These molecular changes are likely due to an imbalance in AC matrix homeostasis resulting from altered cellular activities, which is a consequence of AC injury, thus pointing towards an attempt of the cells and matrix to achieve homeostasis. In general, the observed molecular changes appear more pronounced in regions directly subjected to injury compared to 'kissing' sites.

The relatively high classification accuracy (85%) obtained with an RF-based model for discriminating between the blunt and sharp grooved group (Fig. 5) indicates that these groups are quite distinct in terms of their spectroscopic characteristics (Fig. 4). The moderate injuries (blunt grooved) displayed pronounced spectroscopic characteristics compared to the mild injuries (sharp grooved). The explanation for this phenomenon stems from the fact that both groups of injuries elicit unique molecular alterations in the AC. Blunt injuries typically cause broader and less severe molecular changes, whereas sharp injuries cause more focused and noticeable alterations [34, 50]. The variations are noticeable in the MIR spectral data (Fig. 4), facilitating the differentiation between the two types of injury by machine-learning algorithms. The classification model shows moderate to high accuracy and precision in distinguishing between the different groups of injuries and the control group. The models also performed well in distinguishing between the control and damaged groups, which is a clinically relevant comparison. This indicates that degradation of AC induced by injuries can be sensitively detected using MIR spectroscopy. However, some comparisons, such as between the kissing-blunt group and the kissing-sharp group, showed lower accuracy and precision, suggesting that these groups may have more similar spectroscopic characteristics. While our results align with previous research and histological findings, some comparisons, like between kissing-blunt and kissing-sharp groups, showed subtler differences, indicating a less pronounced variability in injury severity. Overall, these results demonstrate that MIR spectroscopy coupled with ML algorithms can enable the identification of different AC groups.

The regression results showed that MIR spectroscopy is capable of estimating the functional (thickness and biomechanical properties) properties of AC with relatively low error (Fig. 6, Table 2).

Prediction of these physical properties is likely due to an indirect relationship with the composition of AC, which inherently determines the functional properties of the tissue (structure-function relationship). The results from this study further indicate that MIR spectroscopy has the potential to provide valuable information on AC health, including thickness and mechanical properties, which could aid in the diagnostic assessment of AC integrity during repair surgeries. The relatively low RMSEP values indicate that the regression models were able to estimate these parameters from the MIR spectrum reliably.

The regression models for predicting the full-thickness PG content from the MIR spectra are consistent with previous studies [51–54], which have shown the effect of changes in joint contact force and the biomechanical environment on PG depletion near grooves and lesions. The RMSEP of 15.1% for the bluntly grooved group suggests that MIR spectroscopy, which is capable of detecting changes in cartilage PG content, may provide a useful tool for minimally invasive monitoring of AC health and predicting the progression of AC degeneration in patients with joint injuries. Further investigations are warranted to validate the effectiveness of MIR spectroscopy in clinical settings and to explore the potential of this technique for developing personalized treatment plans for individuals with joint injuries. The significant difference in full-thickness PG content between the grooved and control groups validates that the groove model may be useful for studying degenerative changes in AC caused by injuries, as demonstrated by previous studies [16]. These findings highlight the impact of changes in the biomechanical environment of the joint on PG integrity and content near grooves and lesions and suggest that MIR spectroscopy may provide a viable tool for assessing AC integrity during surgery.

However, it is important to acknowledge that this study has limitations. While animal models provide insights, they may not completely replicate the intricacies of injuries in humans and their various forms. Additionally, the study mainly focused on types and severities of injuries, which could potentially restrict the applicability of the findings to a range of AC injuries. Lastly, although the combination of MIR spectroscopy and ML algorithms showed results, it is crucial to consider that AC is inherently complex, and there may be confounding factors in settings that could affect prediction accuracy. Future studies will aim to improve the robustness and generalizability of our methodology, and reduce the variability in error rates, particularly noting that individual predictions can exceed our average error estimate. This observation underlines the necessity for ongoing refinement of our predictive models. Despite these limitations, our study offers insights into how MIR spectroscopy can be used to assess AC injuries and suggests areas for further research in this field.

Our study underscores the potential of MIR spectroscopy, combined with machine learning, as a non-invasive diagnostic tool that could complement existing methods for evaluating cartilage integrity in vivo. By demonstrating the technique's ability to differentiate between types of cartilage injury and assess severity, we highlight a pathway toward real-time, minimally invasive assessment of joint health. This could greatly aid in early detection and monitoring of cartilage degradation, potentially informing treatment decisions and improving patient outcomes. While our findings provide a foundational understanding, it is essential to conduct subsequent in vivo studies to validate these results and refine the methodology for clinical application. Our work serves as a stepping stone toward realizing the full potential of spectroscopic techniques in orthopedic diagnostics.

### Supplementary Information

Below is the link to the electronic supplementary material.

Supplementary file1 (DOCX 1001 KB)
