# Supplementary material for: Infrared Spectroscopy Can Differentiate Between Cartilage Injury Models: Implication for Assessment of Cartilage Integrity
Source: Ann Biomed Eng. 2024 Jun 20;52(9):2521–33. doi: 10.1007/s10439-024-03540-x (PMC11329391; doi:10.1007/s10439-024-03540-x)
Supplement: Supplementary file 1 — Supplementary file1 (DOCX 1001 KB) [file 10439_2024_3540_MOESM1_ESM.docx]

**Supplementary Material**

| 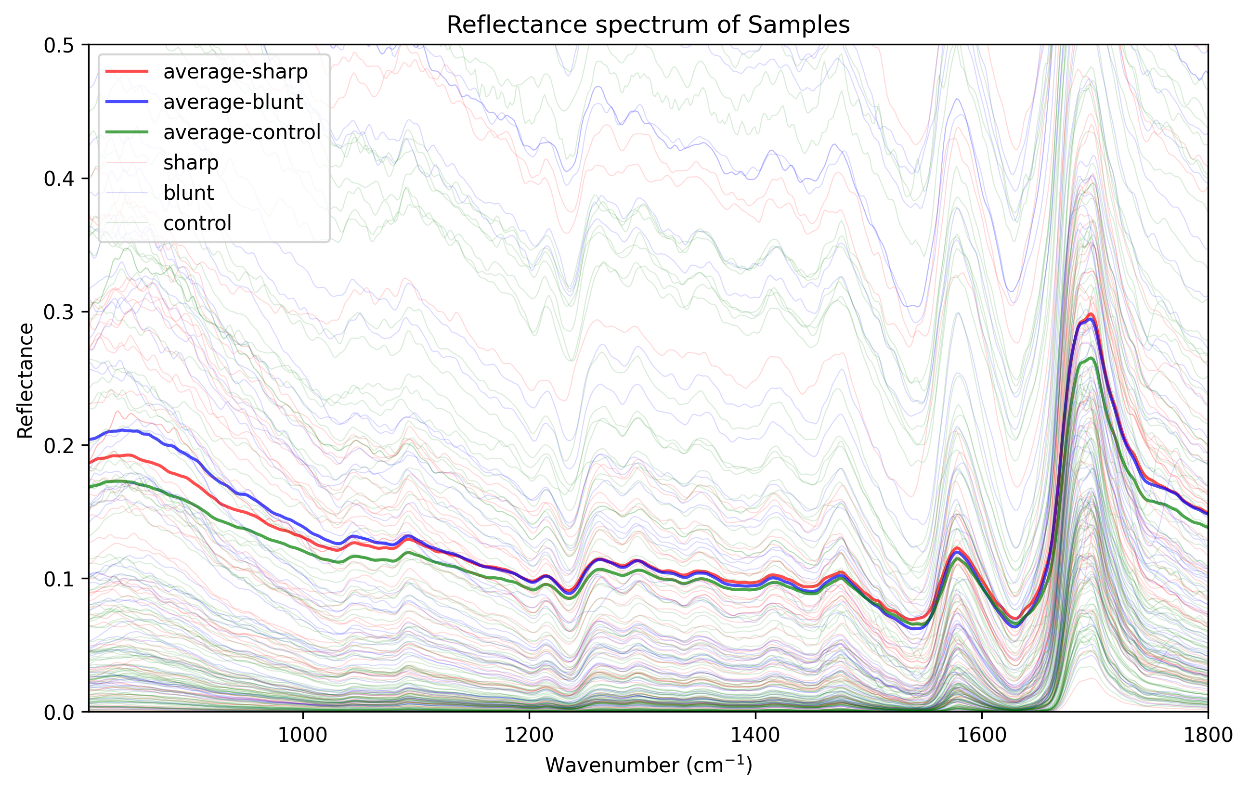 |
| --- |
| *Supplementary 1. Reflectance Spectrum of all groups (Control and Grooved) to visualize and compare the spectral differences directly. The spectra from the kissing sites in this visualization has been excluded to focus on the most significant changes.* |

| **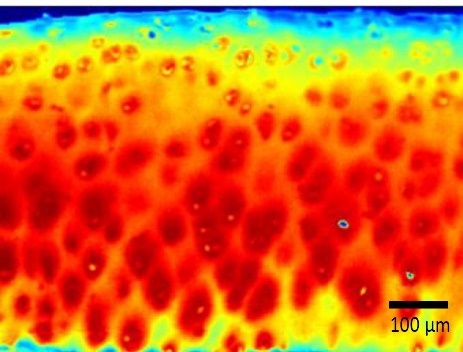** | **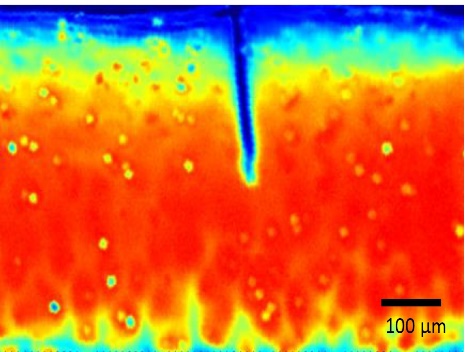** | **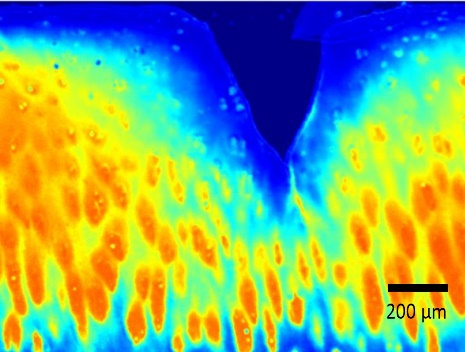** |
| --- | --- | --- |
| **a** | **b** | **c** |
| *Supplementary 2. Representative images of PG content in (a) control,(b) sharp, and (c) blunt grooved cartilage* | | |
